# CRISPR-Cas Technology Turns *Chlamydomonas reinhardtii* into a Flagship for Algal Biotechnology

**DOI:** 10.3390/md24010001

**Published:** 2025-12-19

**Authors:** Amina Antonacci, Annalisa Masi, Vincenzo Vedi, Sara Colella, Federica Musella, Gabriella Fiorentino, Viviana Scognamiglio

**Affiliations:** 1 Institute of Crystallography, Department of Chemical Sciences and Materials Technologies, National Research Council, Via Salaria km 29.300, Monterotondo, 00015 Rome, Italy; amina.antonacci@cnr.it (A.A.); annalisa.masi@cnr.it (A.M.); vincenzovedi@cnr.it (V.V.); saracolella@cnr.it (S.C.); 2 Department of Biology, University of Naples Federico II, Complesso Universitario Monte S. Angelo, Via Cinthia 4, 80126 Naples, Italy; federica.musella2@unina.it (F.M.); fiogabri@unina.it (G.F.)

**Keywords:** CRISPR-Cas, genome editing, microalgae, *Chlamydomonas reinhardtii*, biotechnological applications, added-value compounds

## Abstract

Microalgae represent some of the most promising eukaryotic platforms in biotechnology due to their rapid growth, simple cultivation requirements, reliance on sunlight as a primary energy source, and ability to synthesize high-value bioactive compounds. These characteristics have made microalgae attractive candidates in various fields, including biofuel production, carbon capture, and pharmaceutical development. However, several technical limitations have limited their large-scale use as sustainable biofactories. A paradigm shift is currently occurring thanks to the genetic manipulation of microalgae, driven by CRISPR-Cas technology. Significant progress has been made in the model species *Chlamydomonas reinhardtii*, particularly in the targeted and efficient insertion of foreign DNA. Despite this progress, key challenges remain, and further optimization of CRISPR-Cas methodologies is needed to fully unleash the genetic potential of this organism. This review provides an overview of the convergence of CRISPR-Cas technologies in microalgae research, highlighting their impact on genetic studies, metabolic engineering, and industrial applications. It summarizes recent advances in microalgal genome editing through CRISPR systems, outlines current technical challenges, and highlights future directions for improving the implementation of this innovative technology in microalgal biotechnology.

## 1. Introduction

Microalgae comprise a highly diverse group of species, including *Chlamydomonas* sp., *Synechocystis* sp., *Nannochloropsis* sp., and *Scenedesmus* sp., among many others. These photosynthetic microorganisms possess unique physiological traits that underpin their versatility. For instance, their efficient light-harvesting systems, flexible metabolic pathways, and rapid growth rates allow them to convert sunlight and CO_2_ into biomass with high productivity, reducing the life cycle greenhouse gas (GHG) emissions [1]. They are also capable of accumulating intracellular storage compounds such as lipids, polysaccharides, and proteins, which serve as precursors for biofuels, nutraceuticals/pharmaceuticals, and biodegradable polymers (e.g., bioplastics) [2,3]. Furthermore, microalgae have been described in several studies for their capacity of detoxifying and sensing environmental pollutants, as phenolic compounds and microplastics [4] or pesticides, pathogens, and chemical warfare agents [5]. Many microalgae species have been studied for their potential applications in biotechnology, and improving these organisms for industrial use requires the manipulation of their DNA. In fact, to fully unlock the commercial potential of microalgae, advances in metabolic engineering, strain improvement, and genome editing combined with omics approaches are necessary.

Undeniably, current problems include:

(i) Limited number of microalgal species which can be genetically manipulated, as research focuses only on a few model species;
(ii) Optimization of existing genome-editing technologies and to develop novel genetic tools to enable fine-tuning transgene expression in a temporal and specific manner;
(iii) Challenges concerning genetic stability during the scale-up process [6].

**Figure 1:**
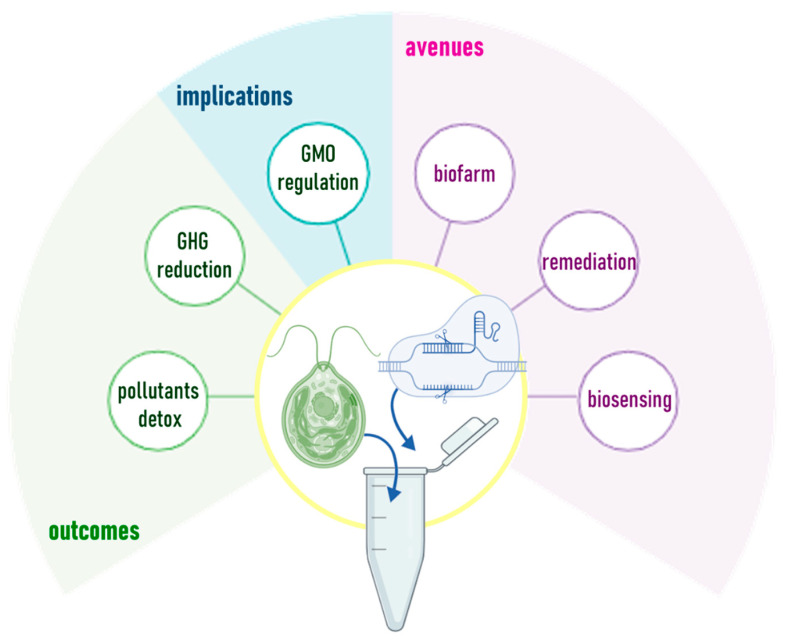
The CRISPR-Cas and microalgae joint revolution with its avenues, implications and results.

Traditional genetic engineering techniques, such as transformation with plasmid vectors, have been reported in the literature; however, these methods often suffer from low efficiency and lack of precision. To this aim, thanks to the recent advent of CRISPR-Cas technology (Clustered Regularly Interspaced Short Palindromic Repeats and CRISPR-associated proteins) the genetic improvement of microalgae has never been so promising (Figure 1). The application of CRISPR-Cas in microalgae research has accelerated the design of genetically modified strains with increased productivity, improved stress resistance, and optimized biochemical pathways [7].

**Figure 2:**
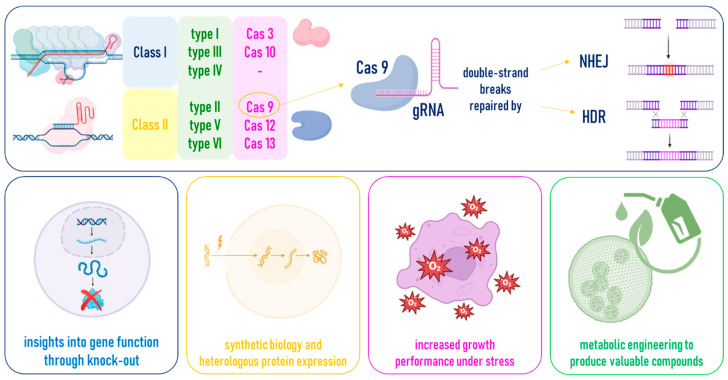
Available CRISPR-Cas systems and their applications in microalgae.

The application of CRISPR-Cas in microalgae entails (Figure 2):

(i) Targeted gene knock-outs, facilitating functional genomics studies or disrupting specific genes involved in key metabolic pathways (e.g., lipid biosynthesis, nitrogen fixation), which can gain insights into the roles of these genes in cellular processes and identify targets for metabolic engineering [8];
(ii) Metabolic engineering to produce valuable compounds of interest for many industrial sectors, such as lipids, polysaccharides, and/or biofuels such as hydrogen [9];
(iii) The introduction of mutations or over-expressions of genes that enhance stress tolerance under different stress conditions, such as high light intensity, nutrient limitation, temperature fluctuations, high salinity or the presence of environmental pollutants, which can be particularly useful in bioremediation applications [10];
(iv) Synthetic biology and heterologous protein expression of foreign genes and pathways, for the production of valuable chemicals for pharmaceuticals, biodegradable plastics, therapeutic proteins, or enzymes for bioremediation [11].

Among the various microalgae, the genus *Chlamydomonas* entails unicellular green organisms with two apical flagella and a cup-shaped chloroplast with a light-sensitive ocellus. These microalgae efficiently perform photosynthesis even in variable environmental conditions, and live in both freshwater and marine environments, with some species exhibiting moderate tolerance to high salinity [12,13]. In recent years, newly discovered extremophilic species have also been identified, capable of thriving in particularly hostile habitats and showing high potential as a biotechnological chassis [14].

Belonging to *Chlamydomonas* sp., *Chlamydomonas reinhardtii* has gained significant attention in recent years thanks to its remarkable properties as a model organism in various biological fields, and to its relatively simple structure, ease of cultivation, and fast growth rate [15]. Its versatility in both autotrophic and heterotrophic growth, along with its ability to express heterologous proteins, positions *C. reinhardtii* as a promising candidate for biofuel production, carbon capture, and synthetic biology. Moreover, the microalgal ability to grow in liquid cultures under a variety of conditions makes it suitable for both laboratory and industrial-scale experiments. Indeed, it is a freshwater microalga, but it showed a moderate ability to tolerate high salinities, likely due to the genetic similarity with marine species [16].

*C. reinhardtii* is also renowned for its robust system for the expression of recombinant proteins, particularly under stress conditions, which has implications for biotechnology and synthetic biology. The ability to manipulate *C. reinhardtii* genome has spurred significant interest in this microalga as a platform for industrial biotechnology. The advent of CRISPR-Cas technology in recent years has revolutionized the possibility to modify *C. reinhardtii* genome, offering unprecedented precision and efficiency for investigating gene function, metabolic pathways, and synthetic biology applications. One of the major advantages of CRISPR-Cas system in *C. reinhardtii* is its ability to bypass the limitations of earlier methods like random mutagenesis or chemical transformation, including low reproducibility, lack of precision in gene targeting or off-target effects, and unpredictable genetic outcomes [17,18]. This will provide further insights into the physiology of the microalga and the potential for developing new strains with industrial implications.

This review aims to explore the synergy between *C. reinhardtii* and CRISPR-Cas technology and their implications in diverse research branches and biotechnological applications. Furthermore, current shortcomings and challenges associated with the use of CRISPR-Cas genome editing in *C. reinhardtii* have been discussed as well as potential future directions for harnessing this powerful combination in biotechnological advancements.

## 2. Progress of CRISPR-Cas Technology in *C. reinhardtii*

*C. reinhardtii* has been widely used as a model organism in various fields of biology. Its simple structure, combined with the presence of one chloroplast and two flagella, makes it ideal for the study of fundamental cellular processes such as photosynthesis, chloroplast biogenesis, mechanisms of movement, assembly of microtubules [19] and the cell cycle [20]. Thanks to its ability to grow in both autotrophic and heterotrophic conditions, it has significantly contributed to the understanding of photosynthesis. In addition, the easy genome manipulation allowed to discover the genes involved in this process [21]. *C. reinhardtii* is also the first microalga to undergo a genome-sequencing project [22,23] and for this reason, it has attracted increasing research actions to develop biotechnological applications in the environmental sector. Thanks to its remarkable versatility, it has been successfully utilized as a biological platform to produce polysaccharides and hydrogen under anaerobic conditions [24], making it a potential candidate for sustainable production of added value compounds as bioplastic or biofuel. Furthermore, *C. reinhardtii* has been exploited in bioremediation strategies and the field of biosensing [25,26].

Latest trends in CRISPR technology are positioning *C. reinhardtii* as a flagship organism in algal biotechnology thanks to its simplicity of genetic manipulation and broad applications in research and industry. CRISPR-Cas system, initially discovered in bacteria as an adaptive immune system, has quickly driven a revolution in manipulative genetics, allowing the development of genetic tools for precise and targeted genome alterations of a wide range of organisms, including microalgae. Such systems are categorized into two major classes based on their structural organization and distinct targeting mechanisms: Class I, which includes types I, III, and IV, and Class II, comprising types II, V, and VI. Class II systems employ a single multidomain effector protein to mediate nucleic acid cleavage, a feature that makes them particularly suitable for gene editing applications due to their simplicity and ease of use. The most widely used system for genetic modification is based on CRISPR-Cas9, which uses a guide RNA (gRNA) to target a specific DNA sequence and employs the Cas9 endonuclease to introduce double-strand breaks at the target site. This last can be then repaired by the cell’s repair mechanisms, through non-homologous end joining (NHEJ), homologous recombination (HR), or homology-directed repair (HDR), which can result in insertions, deletions, or precise gene corrections [27]. In addition to Cas9, other CRISPR-associated proteins, such as Cpf1 (now known as Cas12) and C2c2 (known as Csm/Cmr complexes), have expanded the toolbox of CRISPR technologies, offering variations in target specificity, editing precision, and even RNA-based editing [28].

The application of CRISPR-Cas systems has been firstly reported in *C. reinhardtii* [29] for transient expression and targeted restriction sites of diverse exogenous genes. Subsequently, a high-throughput CRISPR-Cas9 system for *C. reinhardtii* was developed, enabling the editing of multiple gene targets simultaneously and thus demonstrating the potential for using CRISPR-Cas9 for microalgal metabolic engineering [30]. For example, CRISPR-Cas9 was used to engineer *C. reinhardtii* to produce valuable compounds like carotenoids, astaxanthin, and pharmaceutical proteins, demonstrating its potential also as a green “cell factory” for sustainable bioproduction. These pioneering studies clearly showed that “scarless knock-in mutagenesis” in microalgae and especially in *C. reinhardtii* is no longer a mirage but a concrete and operational technology. Indeed, such outcomes will pave the way for further advances in genome engineering, by enabling precise modifications as targeted gene knock-outs, replacements, and insertions, which could in the future contribute to the creation of genetically stable and high-performance strains for industrial applications.

These are just a few examples of the potential of applying CRISPR-Cas technology to *C. reinhardtii* not only to broaden knowledge on cell mechanisms and physiology, but also to provide improved microalgal strains with selected biotechnological implications. In the following chapters, the use of CRISPR-Cas technology in *C. reinhardtii* is discussed according to the diverse physiological advancements and to the various applications.

### 2.1. Harnessing CRISPR-Cas Technology for Advancing C. reinhardtii Genome Editing

From classical mutagenesis to advanced genome editing, *C. reinhardtii* represented a valuable tool for the study of cellular and molecular biology. However, conventional methods have numerous limitations: random mutagenesis lacks target specificity and reproducibility and produces off-target mutations; homologous recombination exhibits low efficiency and limited scalability and requires selectable markers and long homology arms; zinc finger nucleases (ZFNs) are characterized by high off-target activity, low precision, and difficult delivery; and transcription activator-like effector nucleases (TALENs) are laborious and time-consuming, with limited flexibility and lower knock-in efficiency.

**Figure 3:**
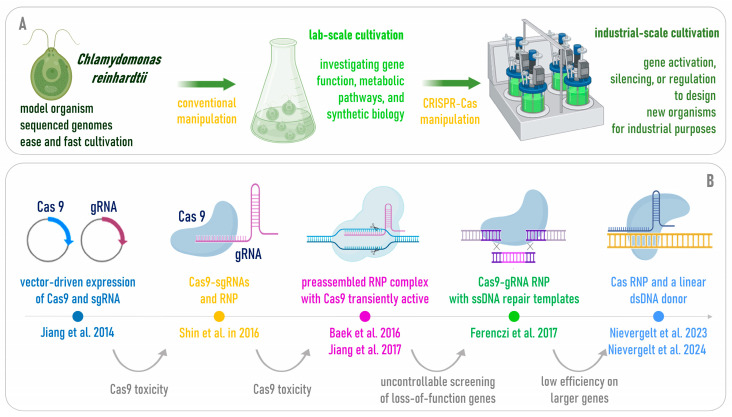
(**A**) CRISPR-Cas systems turn out microalgae from laboratory to industrial biotechnology. (**B**) Evolution of the CRISPR system configurations over the last 10 years from the first study of Jiang et al. [29] to their further improvements described by Shin et al. [38], Baek et al. [39], Jiang et al. [40], Ferenczi et al. [41], and Nievergelt et al. [44,45].

Conversely, the CRISPR-Cas system turned out to be a simpler, versatile, and trustable technique to edit the microalgal genome to study and improve its mechanisms, as well as knock-in and out genes of interest for various bioengineering (Figure 3A).

As above mentioned, the first study reporting the use of CRISPR-Cas technology to engineer *C. reinhardtii* dates back to 2014 [29]. The authors adapted a system previously optimized for plant protoplasts and leave to express the Cas9-single guide RNA (sgRNA) in *Chlamydomonas* using a codon-optimized Cas9 gene. Even though foreign genes can be easily integrated into the microalga, transgene expression is often weak due to differences in GC content in *C. reinhardtii* codons that drastically influences gene expression at the chromatin level [31,32,33,34,35,36,37]. In this case [29], the authors were unable to select cells carrying targeted gene modifications claiming the possibility that Cas9 expression could be toxic. Therefore, considering the possibility that the continuous expression of Cas9 could be toxic, a transient vector-driven expression of Cas9 and sgRNA genes was carried out resulting in precise targeting of four independent target genes. In detail, the exogenously supplied genes (hygromycin resistance, gfp, and Gluc) and the endogenous fkb12 (rapamycin sensitivity) gene of *C. reinhardtii* displayed distinct Cas9-sgRNA-mediated target site modifications. However, only one rapamycin-resistant colony bearing an appropriately modified FKB12 target site was recovered in 16 transformation experiments involving >10^9^ cells, highlighting the low efficiency of the system.

Following the extremely low targeting efficiency shown by this latest study, possibly still resulting from the toxicity induced by vector-driven Cas9 expression, Shin et al. [38] tried to directly deliver the Cas9-sgRNAs ribonucleoprotein (RNP) in the cells, instead of cloning their genes in a vector. The new system was used to target three genes: the maa7 gene encoding the beta subunit of tryptophan synthase, the antennal assembly gene cpSRP43 encoding chloroplast SRP43, and the chlorophyll biosynthetic gene chlM encoding Mg-protoporphyrin IX S-adenosyl methionine O-methyl transferase. Co-transformation of vectors conferring hygromycin resistance was also provided for the exogenous genes. This new system allowed to verify mutations occurring to all the selected genes in proximity of the expected Cas9 cleavage site. However, with this single exception, the proposed RNP delivery system failed to produce viable cells with numerous other endogenous targeted gene modifications. Toxicity due to Cas9 continuous expression in transformed cells continued to represent a possible failure to recover cells containing intact Cas9 genes. Thus, while Cas9 genes and sgRNAs were able to promote targeted genome editing in *Chlamydomonas*, the recovery of stable edited cells was very low, if not absent.

The potential toxicity of stably expressed Cas9 was once again reported [38], emphasizing the importance of a system limiting its accumulation Cas9 in transformed cells and suggesting the use of conditional promoters to drive Cas9 gene expression or transformation, with short-lived Cas9 mRNAs as one of the viable strategies. In the following years, diverse studies demonstrated how the transformation of *Chlamydomonas* cells with a preassembled RNP complex has reduced toxicity, as Cas9 protein was transiently active and then degraded by endogenous proteases in cells [39,40]. Moreover, increased on-target efficiencies occurred and codon optimization of the cas9 gene was avoided. For example, Baek et al. [39] described the use of DNA-free RGENs, i.e., preassembled Cas9-gRNA ribonucleoproteins, which eliminated the need for specific codon or promoter optimization in several species, including *C. reinhardtii*. Jiang et al. [40] further improved the preassembled RNP complex for the transient Cas9 expression by designing a hybrid gene-within-a-gene construct constituted by a cas9 gene containing an artificial intron with an inserted sgRNA gene, to reduce the number of genes needed from two (i.e., cas9 and sgRNA genes) to one. The authors demonstrated that this construct was sufficient for most gene editing projects in *Chlamydomonas* although the gene editing efficiency was not high.

Subsequently, the use of selectable markers by co-targeted insertion and co-delivery of RNPs with ssDNA repair templates facilitated the screening of genes whose loss-of-function mutation caused visible and known phenotypes [41]. Even later, mutants for genes with non-selectable phenotypes were obtained by co-selection with exogenous antibiotic-resistant cassettes or by mutation of an endogenous gene that produced a dominant selectable phenotype [42]. These studies have highlighted the importance of using not only Cas9-gRNA RNPs but also repair templates for gene editing. However, the efficiency of mutant isolation for genes with low transcription levels remains low and nonspecific. Furthermore, precise genetic manipulation, such as the insertion of DNA sequences encoding an epitope or a fluorescent tag, or the replacement of an amino acid at a desired site, was not yet straightforward and there was a strong demand for new approaches. To this aim, strategies including homology-directed repair and microhomology-mediated end-joining (MMEJ) helped to obtain predictable insertion of donor DNA but their efficiency in *Chlamydomonas* remained low [43]. This type of mutant is rescued by introducing a recombinant copy of the altered gene. However, rescue experiments have proven difficult for larger genes, because these insertions might randomly interrupt an unknown part of the genome, and donor templates tend to form secondary structures that might inhibit efficient transformation. To overcome these challenges, Nievergelt et al. [44] set up a protocol for homology-directed knock-in mutagenesis for large functional inserts, even fusing them with resistance cassettes, obtaining higher efficiency and robust recombinant lines over time. In detail, they provided an efficient homology-directed method for knockin mutagenesis in *Chlamydomonas* by delivering a CRISPR-Cas ribonucleoproteins and a linear double-stranded DNA (dsDNA) donor, allowing for scarless integration of fusion tags and sequence modifications of proteins without the need for a preceding mutant line. This method, further optimized in 2024 [45], produced good results in inserting long insertions, and almost all insertions resulted in perfect HDRs. Therefore, it was not only useful for gene disruption in *Chlamydomonas* but also for functional knock-in modifications for endogenous tagging. However, these studies not considered that a successful gene editing could still result in a non-functional protein or changes in expression levels due to misfolding or changes in regulatory elements. In this case, lethal gene editing could result in no or fewer offspring. Furthermore, these studies have not targeted chloroplast or mitochondrial genes to date.

Despite the evolution of the CRISPR system configurations over the last 10 years (Figure 3B), the poor efficiency of CRISPR-based genome editing could also be ascribed to the delivery systems of the components, which may be hampered by constraints due to the cell wall. Available transformation methods include electroporation, biolistic transformation, and Agrobacterium-mediated one. To try to improve the delivery of Cas9/sgRNA complex into microalgal cells, Kang et al. [46] described the use of the cell penetrating peptide pVEC (LLIILRRRIRKQAHAHSK) derived from the murine vascular endothelial cadherin protein and working in a non-covalent form. As an alternative to electroporation, this method turned out to be simple and time-saving (~30 min) and no bulky equipment was required. Witman’s group [47,48] designed a delivery technique based on the co-use of the Cas9-gRNA ribonucleoprotein together with exogenous (donor) double-stranded DNA containing gene-specific homology arms and a full-length antibiotic resistance gene that fits into the double-strand break generated by Cas9. This method, called TIM [47], introduces Cas9-sgRNA ribonucleoproteins and a linear donor DNA cassette into *C. reinhardtii* using cell wall removal (autolysin treatment) followed by electroporation. This combination greatly enhances membrane permeability and nuclear delivery compared to traditional approaches such as glass-bead agitation. Donor DNA is then integrated at Cas9-induced double-strand breaks via NHEJ, enabling efficient and targeted knock-in mutagenesis. The optimized procedure resulted in mutation efficiencies ranging from 40 to 95% and in simultaneous targeting of two separate genes in a single experiment.

As an alternative to the Cas9 protein, the CRISPR system was designed for *C. reinhardtii* also using the Cas12 protein. Ferenczi’s group [49,50] provided a comprehensive picture of the applicability of Cas9 and Cas12a in single-stranded DNA repair and gene editing by mapping their targeting space (analyzing the corresponding PAM frequencies) and then comparing the activities of these two components by targeting overlapping regions at three independent loci in the *Chlamydomonas* genome. The results showed that Cas9 and Cas12a RNPs co-delivered with ssODN repair templates were able to induce similar levels of total editing, with slightly higher accuracy for Cas12a. On the contrary, Cas9 alone delivered more edits to the FKB12 locus than Cas12a, making it the preferred enzyme for genome engineering among currently available nucleases.

Noteworthy, the literature regarding CRISPR-Cas technology in the last 10 years has provided many tools for gene editing and knock-out and is growing exponentially to offer other configurations to overcome the main limitations, such as nonspecific mutagenesis and low efficiency. However, this research remains in its infancy, and currently only gene editing and knock-out of genes for altering enzymatic pathways (e.g., to enhance lipid or pigment production) have been widely described. Advances in scarless knock-in of exogenous DNA fragments (e.g., genes for antibiotic resistance) have also been described for their controlled insertion into the *Chlamydomonas* genome, but not for full-length genes encoding enzymes to be heterologously overexpressed to provide novel functions to the microalga. A very recent example has been reported by Zadabbas et al. [51], which applied the homology-directed DNA repair CRISPR-Cas9-mediated knock-in to insert by homologous recombination a bacterial phytase gene at a specific site of the nitrate reductase (NR) gene (exon 2) of *C. reinhardtii*. A bacterial phytase gene cassette was directly delivered through a ribonucleoprotein complex consisting of Cas9 protein and the specific sgRNAs. The editing efficiency was determined to be 14.81%, the stability of correct editing without gene silencing or negative insertion was observed for 10 generations of knock-in positive colonies, and the site-specific nuclear gene expression did not show nuclear positional effects and gene silencing. These findings provide a new perspective on the beneficial application of RNP-based CRISPR-Cas9 gene editing to accelerate the commercial production of complex recombinant proteins in *C. reinhardtii*, which may find countless biotechnological applications.

Considering all studies conducted since the first use of CRISPR technology in *C. reinhardtii* in 2014 [29] to date, we can conclude that the evolution of gene editing has shifted towards the RNP-based CRISPR-Cas9 system to circumvent the cytotoxicity constraints and low recovery rates associated with continuous vector-based Cas9 expression. Using the RNP complex, transient nuclease activity can be achieved, maximizing the editing window and minimizing cellular stress. However, despite this technical refinement, low editing efficiency remains, especially for complex modifications. This low yield requires continued reliance on selectable markers for the isolation of engineered lines, complicating scar-less gene integration and limiting scalability for high-throughput synthetic biology. Furthermore, research focuses primarily on nuclear edits (e.g., endogenous gene knockout), leaving the chloroplast and mitochondrial genomes completely unexplored. Because the chloroplast hosts essential metabolic pathways for photosynthesis and the synthesis of high-value products (e.g., lipids and pigments), this exclusion now represents a vast genomic resource. Therefore, a further priority is to fully exploit the potential of *C. reinhardtii* as a robust biofactory.

### 2.2. CRISPR-Cas in C. reinhardtii: From Elucidating Cellular Mechanisms to Unlocking the Potential for Sustainable Biomanufacturing

Microalgae, with *C. reinhardtii* as a model organism, show high potential to produce valuable compounds for use in various biotechnological applications, including proteins, polysaccharides, pigments, and lipids, among others. However, to date, low productivity and high production costs have restricted their commercialization. Despite targeted efforts, it has been shown that ad hoc modifications of growth conditions have not consistently resulted in enhanced productivity. For this reason, in recent years genetic engineering has become increasingly necessary to obtain microalgal strains specifically designed for specific industrial applications.

As above mentioned, random mutagenesis, homologous recombination, zinc finger nucleases, and transcription activator-like effector nucleases, among others, show many shortcomings. To this end, the CRISPR-Cas tool has been instrumental in precise and targeted genetic modification and modulation of gene expression. This allowed not only to elucidate many cellular mechanisms to enhance the microalgal performances in stress conditions, but also to project new industrially relevant strains with improved productivity and photosynthetic capacity.

For example, Findinier et al. [52] investigated the role of the dynamin-like protein Fzl, which is crucial for promoting thylakoid membrane fusion, a key process for maintaining the integrity of the photosynthetic apparatus under stress conditions. To explore its function, CRISPR-Cas9 technology was employed to generate multiple knock-out strains, revealing a specific requirement of CrFzl for survival upon light stress.

With a similar system, Jeong et al. [53] investigated the role of the chloroplast protein LTD in the assembly of the light-harvesting complex (LHCI) and photosystem I (PSI) in *C. reinhardtii*. By deleting the gene encoding LTD, they observed a significant disruption in the import of LHCI subunits into the chloroplast and a failure in PSI-LHCI assembly.

Kim et al. [54] explored the impact of a knock-out of the β-carotene hydroxylase gene on the xanthophyll content and growth of *C. reinhardtii*, whose enzyme is involved in the conversion of β-carotene to xanthophylls. By knocking out this gene, a partial reduction in xanthophyll levels was observed, which led to enhanced high-density cultivation of *Chlamydomonas*. The reduced xanthophyll content helped to improve the microalgae biomass production under conditions typically used for mass cultivation, making the process more efficient.

Asadian et al. [55] utilized the CRISPR-Cas9 technique to knock-out the cia5 gene in *C. reinhardtii*, implicated in the regulation of CO_2_ concentration mechanisms. The effects of this genetic alteration on CO_2_ sequestration capabilities showed that the cia5 knock-out mutants exhibited altered CO_2_ uptake and fixation compared to the control strains, highlighting the potential for genetic modifications to optimize CO_2_ utilization for applications, e.g., in carbon capture.

Shimamura et al. [56] investigated the role of the periplasmic carbonic anhydrase enzyme CAH1 in *C. reinhardtii* and its contribution to high inorganic carbon affinity. The results showed that CAH1 is involved in enhancing the microalgal ability to capture and utilize inorganic carbon, particularly under low-carbon conditions. The findings highlighted the critical role of CAH1 in the carbon concentrating mechanism, enabling *Chlamydomonas* to thrive in environments with limited carbon availability, providing important insights into the molecular mechanisms that contribute to carbon uptake and fixation, with potential applications in enhancing biofuel production and carbon sequestration technologies.

Besides the enhancements of the performances of the cellular mechanisms, CRISPR-Cas systems enabled in the last few years to design novel *Chlamydomonas* strains capable of overproducing added value compounds for diverse applications, from nutraceutics to biofuel. As an example, Nguyen et al. [57] explored the use of CRISPR-Cas9 technology to enhance lipid productivity in *C. reinhardtii* for biofuel production, modulating lipid catabolism and boosting their accumulation. Specific genes involved in lipid degradation were knocked-out, thus causing a significant increase in lipid content, with an increase of 28% of the dry biomass compared to 22% of the wild type, without affecting the overall growth of the algae.

Similar studies reported in the last few years showed that lipid accumulation can be mediated through the regulation of phosphoenolpyruvate carboxylase enzyme [58] or by knock-out of genes involved in lipid metabolism. Crucial examples are also those encoding phospholipase A2 [59], DGTT1-3, PDAT [60], and carboxyltransferase interactor 1 (CTI1) [61].

Pigment content has also been improved in *Chlamydomonas* by the modification of their biosynthesis pathways. Baek et al. [62] genetically modified *C. reinhardtii* with DNA-free CRISPR-Cas9 technology to increase the production of carotenoids lutein and zeaxanthin, known for their protective role against age-related macular degeneration. The *C. reinhardtii* CC4349 cell line, considering as the most promising among seven strains tested for carotenoid production, was treated with preassembled DNA-free CRISPR-Cas9 ribonucleoproteins to create a mutant with the inactivated zeaxanthin epoxidase gene. This mutant showed significantly higher zeaxanthin content (56-fold) and higher productivity (47-fold) than the wild type, without a reduction in lutein levels. In addition, by feeding hens a diet containing the mutant, the authors were able to produce eggs enriched with lutein (2 times) and zeaxanthin (2.2 times), demonstrating the potential commercial application of microalgal mutants for the production of high-value products.

Song et al. [63] created a double knock-out by CRISPR-Cas9 RNP-mediated knock-in targeting the lycopene epsilon cyclase encoding gene (LCYE) and using the ZEP mutant as a parental line to inhibit the biosynthesis of α-carotene. This approach allowed for a 60% higher zeaxanthin yield.

Kneip et al. [30] explored the use of CRISPR-Cas9 technology to inhibit the lycopene ε-cyclase (LCYE) gene in *C. reinhardtii* microalgae. The knock-out of the LCYE gene reduces the synthesis of α-carotene and its derivatives, redirecting metabolic flow to the production of β-carotene and astaxanthin, a commercially valuable carotenoid. The mutant ΔLCYE#3, obtained through this approach, showed a significant increase in astaxanthin production, with growth comparable to that of the parent UVM4 strain under normal light conditions. The reduction of α-carotene-related carotenoids improved efficiency in astaxanthin production, with a 44% increase in astaxanthin yield compared to the wild-type strain. Overexpression of key enzymes further enhanced astaxanthin biosynthesis. The knock-out of the LCYE gene led to increased availability of precursors for astaxanthin production without impairing cell growth, confirming the potential of *C. reinhardtii* as a platform for the sustainable production of high-value carotenoids for commercial use.

Yan et al. [64] focused on engineering *C. reinhardtii* for enhanced glycolate biosynthesis, targeting the hydroxypyruvate reductase 1 (HPR1) gene using a knock-out strategy, to disrupt the conversion of hydroxypyruvate to glycerate and redirect carbon flow toward glycolate production. In addition to the genetic modification, culture conditions were optimized to further boost glycolate production. The results showed that the combination of the HPR1 knock-out and optimized growth conditions led to a significant increase in glycolate accumulation, useful in bio-based chemicals and sustainable production processes.

It is clear that C. reinhardtii offers great potential for the production of valuable compounds (e.g., proteins, lipids, pigments), yet its commercial viability remains hampered by low productivity and high costs. These latest studies, reported in Table 1, have demonstrated that targeted CRISPR-Cas genome engineering may become essential to overcome the limitations of inefficient ad hoc environmental modulation and outdated genetic methods. CRISPR’s main strengths have been its ability to (i) facilitate mechanism-based functional studies that inform strain optimization and (ii) enable the rational reengineering of metabolic pathways to scale up industrial production.

Despite these studies validate CRISPR-Cas as a necessary tool for rational strain development, current attention remains focused on knockout strategies to simply remove existing metabolic bottlenecks. Future efforts should prioritize precise knockout and complete pathway integration, leveraging the microalgae’s enhanced photosynthetic capacity to design truly innovative, high-throughput biosynthetic pathways, thus moving beyond simple genetic fine-tuning to the construction of a complete biofactory.

## 3. Future Directions and Conclusions

The emergence of CRISPR-Cas genome editing has markedly transformed microalgal biotechnology, positioning *C. reinhardtii* as a powerful photosynthetic chassis for synthetic biology, metabolic engineering, and industrial applications. Compared with classical mutagenesis, CRISPR-Cas systems offer unprecedented programmability, efficiency, and versatility in altering nuclear, chloroplast, and potentially mitochondrial genomes. In recent years, the deployment of CRISPR-Cas9, Cas12a, and engineered high-fidelity nucleases has enabled not only targeted gene knockouts via non-homologous end-joining, but also gene regulation, scarless knock-in mutagenesis, and multiplexed genome manipulations that were previously inaccessible in this organism.

Nevertheless, despite these significant advances, CRISPR editing in *C. reinhardtii* remains technically challenging. A fundamental bottleneck is the delivery of CRISPR components (e.g., Cas proteins, guide RNAs, and repair templates) across the robust glycoproteinaceous cell wall characteristic of many strains. While cell wall-deficient mutants (e.g., cw15, CC-503) may facilitate electroporation-based delivery, these strains often exhibit altered physiology and reduced environmental resilience. Newer approaches, including nanoparticle-mediated uptake, lipid vectors, viral systems, or RNP complexes, show promise but require further optimization to achieve reproducibility across strains and growth conditions.

A second major limitation concerns Cas-associated cytotoxicity. Persistent expression of Cas9 can lead to growth retardation, DNA damage responses, and reduced viability, motivating the development of inducible promoters, high-fidelity variants with reduced off-target activity, and transient RNP-based approaches that minimize nuclease exposure. Importantly, off-target cleavage remains a significant concern. Several studies indicate that Cas9 in microalgae exhibits tolerance to mismatches within the guide RNA, increasing the likelihood of unintended mutations.

Equally challenging is the inefficiency of precise knock-in events mediated by homology-directed repair. In contrast to yeast or mammalian cells, *C. reinhardtii* strongly favors NHEJ, which readily produces insertion/deletions but only rarely supports the insertion of large genetic cassettes or full-length open reading frames. Although strategies such as Targeted Insertional Mutagenesis, donor DNA optimization, and cell-cycle synchronization have improved HDR rates, the integration of complete metabolic pathways, rewiring of photosynthetic modules, or construction of synthetic biosynthetic clusters remains considerably constrained. Emerging precision-editing tools such as base editors and prime editing have not yet reached routine applicability in microalgae, but hold potential to sidestep the reliance on HDR.

As the field matures, expanding CRISPR-Cas systems beyond *C. reinhardtii* remains a central priority. Species such as *Nannochloropsis*, *Phaeodactylum tricornutum*, *Dunaliella salina*, *Haematococcus pluvialis*, and *Chlorella* spp. offer unique metabolic features, including valuable bioactive compounds, which could serve as alternative scaffolds for industrial and pharmaceutical applications, but lag behind in genome-editing tools. Developing robust genetic systems also towards these microalgae will broaden the applicability of CRISPR technologies and unlock new routes for accelerating functional genomics and identifying novel genes and pathways of biotechnological interest.

Finally, it is increasingly recognized that ethical, regulatory, and biosafety considerations will shape the future use of genome-edited microalgae. The environmental release of engineered phototrophs raises questions about horizontal gene transfer, ecosystem perturbation, selective advantages under changing climate scenarios, and the persistence of edited genomes in natural communities. Moreover, regulatory frameworks vary across jurisdictions, and the distinction between “genome-edited” and “genetically modified” organisms remains ambiguous in many regions. Harmonized guidelines are urgently needed to ensure responsible research, transparent risk assessment, and public acceptance.

Looking ahead, fully exploiting the potential of CRISPR-Cas technology in *C. reinhardtii* and other microalgae will require significant advances in both molecular tools and cultivation platforms. It will be crucial to develop more efficient and less traumatic delivery methods, also applicable to strains with intact cell walls, along with high fidelity, low-cytotoxic nucleases capable of ensuring more precise editing. At the same time, improved control of DNA repair mechanisms will be necessary to enable the insertion of complete genes and complex metabolic modules, as well as counteracting epigenetic silencing phenomena that still limit the stability of engineered lines. Expanding CRISPR applications to non-model microalgal species will open new opportunities for the sustainable production of high-value metabolites, biofuels, and bioactive molecules, but will require systematic approaches to metabolic modeling, selection of “safe-harbor” *loci*, and optimization of transgene expression. At the same time, the transition to industrial applications requires integrating sustainability, biosafety, and regulatory governance assessments from the early stages of development. Only through the parallel advancement of editing technologies, robust biological chassis, standardized protocols, and clear regulatory frameworks will it be possible to transform microalgae into reliable, scalable biofactories compatible with the principles of the circular economy and environmental sustainability.

## Figures and Tables

**Table 1:** CRISPR-Cas editing in *C. reinhardtii* for gene role investigation as well as enhanced cultivation performances and bioproduction.

Editing Configuration	Outcome	Ref.
Multiple knock-out, revealing insights for survival upon light stress	Protein Fzl role evidenced, marker of photosynthetic apparatus integrity under stress conditions	[52]
Knock-out of the LTD encoding gene, revealing failure in light-harvesting complex (LHCI)—photosystem I (PSI) assembly	Chloroplast protein LTD role evidenced in the assembly of the LHCI and PSI	[53]
Knock-out of the β-carotene hydroxylase gene	Reduced in xanthophyll levels that led to enhanced high-density cultivation and improved biomass	[54]
Knock-out of cia5 gene implicated in the regulation of CO_2_ concentration mechanisms	Altered CO_2_ uptake and fixation, with potential for carbon capture regulation	[55]
Knock-out of periplasmic carbonic anhydrase enzyme CAH1	CAH1 contribution to capture and utilize inorganic carbon	[56]
Knock-out of genes involved in lipid degradation	Enhanced lipid productivity for biofuel production, boosting lipid accumulation	[57,58,59,60,61,62]
Knock-out and Knock-in of genes involved in pathways for pigment production	Enhanced yield of pigment content, e.g., zeaxanthin and astaxanthin	[30,61]
Knock-out of the hydroxypyruvate reductase 1 (HPR1) gene	Increased glycolate accumulation, useful in bio-based chemicals and sustainable production processes	[64]

## Data Availability

Not applicable.
